# A Comprehensive Review on the Processing of Dried Fish and the Associated Chemical and Nutritional Changes

**DOI:** 10.3390/foods11192938

**Published:** 2022-09-20

**Authors:** Nursyah Fitri, Sharon Xi Ying Chan, Noor Hanini Che Lah, Faidruz Azura Jam, Norazlan Mohmad Misnan, Nurkhalida Kamal, Murni Nazira Sarian, Mohd Aizuddin Mohd Lazaldin, Chen Fei Low, Hamizah Shahirah Hamezah, Emelda Rosseleena Rohani, Ahmed Mediani, Faridah Abas

**Affiliations:** 1 Department of Biosciences, Faculty of Science, Universiti Teknologi Malaysia, Johor Bahru 81310, Malaysia; 2 Institute of Systems Biology (INBIOSIS), Universiti Kebangsaan Malaysia UKM, Bangi 43600, Malaysia; 3 Faculty of Medicine, Manipal University College Malaysia (MUCM), Jalan Padang Jambu, Bukit Baru 75150, Malaysia; 4 Herbal Medicine Research Centre, Institute for Medical Research, National Institutes of Health, Shah Alam 40170, Malaysia; 5 Department of Food Science, Faculty of Food Science and Technology, Universiti Putra Malaysia, Serdang 43400, Malaysia

**Keywords:** fish, drying, nutritional compositions, omics, sensory attributes, microorganisms, safety

## Abstract

Fish is a good source of nutrients, although it is easily spoiled. As such, drying is a common method of preserving fish to compensate for its perishability. Dried fish exists in different cultures with varying types of fish used and drying methods. These delicacies are not only consumed for their convenience and for their health benefits, as discussed in this review. Most commonly, salt and spices are added to dried fish to enhance the flavours and to decrease the water activity (a_w_) of the fish, which further aids the drying process. For fish to be dried effectively, the temperature, drying environment, and time need to be considered along with the butchering method used on the raw fish prior to drying. Considering the various contributing factors, several physicochemical and biochemical changes will certainly occur in the fish. In this review, the pH, water activity (a_w_), lipid oxidation, and colour changes in fish drying are discussed as well as the proximate composition of dried fish. With these characteristic changes in dried fish, the sensory, microbial and safety aspects of dried fish are also affected, revolving around the preferences of consumers and their health concerns, especially based on how drying is efficient in eliminating/reducing harmful microbes from the fish. Interestingly, several studies have focused on upscaling the efficiency of dried fish production to generate a safer line of dried fish products with less effort and time. An exploratory approach of the published literature was conducted to achieve the purpose of this review. This evaluation gathers important information from all available library databases from 1990 to 2022. In general, this review will benefit the fishery and food industry by enabling them to enhance the efficiency and safety of fish drying, hence minimising food waste without compromising the quality and nutritional values of dried fish.

## 1. Introduction

Fish are usually dehydrated as a means of preservation, as fish is well-known for its perishability issue. For regions that have easy access to large areas of water, fish is a major source of nutrients for their community. In addition, dried fish differs from place to place and culture to culture, from the type of fish used to the drying methods and ingredients used in the drying process. Stockfish and clipfish are examples of dried fish products from Norway [1,2]. On the other hand, Asian countries like China, Bangladesh, and Japan are also significant contributors to the dried fish industry [3]. Stockfish is an unsalted dried fish product made by air drying Arctic cod (*Gadus morhua*), specifically ones migrating Northeast [1]. In Norwegian cultures, the cod are beheaded, gutted, and hung in the cold open air (0–2 °C) to dry for 3 months [1]. Then, the dried fish is stored for 2–12 months to mature before they are shipped to be sold. Interestingly, other types of white fish, such as pollock, haddock, ling, and tusk are also used to make stockfish aside from the traditional and most commonly used cod [4]. Although the product originates from Norway, its consumption has spread worldwide due to large exports. In 2018 alone, Norway exported 5200 tonnes of stockfish to mainly Italy and other countries like Nigeria, Sweden, and France [1].

Other than stockfish, Norway also mass produces clipfish, which is essentially salted dried fish. Similar to stockfish, clipfish is made using Norwegian cod. Traditionally, the fish is disembowelled and left to air-dry in harsh, unpredictable weather conditions. However, modern studies have developed more efficient methods to dry the fish, especially convection [2]. Before the fish is dried, salt is added to the fish for maturation. For ideal osmotic dehydration to occur, the head and backbone of the fish are removed, and the remaining flesh is folded in a specific way before it is covered with salt for 3–4 weeks [2]. In contrast to air-drying, Chinese cultures prefer sun-drying fish, especially seawater fish. However, most vendors or households casually dry the fish in the sun, exposed to the environment [5]. This preference is also seen in Bangladesh and India [6,7]. This might be influenced by the low cost of the drying method, as the widely practised sun-drying method is utilised by people on all economic levels. Additionally, the fish are cured with salt to cater to the flavour preferences of the market and prevent microbial growth [6]. The fishes used to make dried fish vary depending on the seasonal local fish populations. For instance, Loitta and Kanchki are commonly used in Bogra region of Bangladesh, while Churi, Chingri, Hangor and Chanda are used in other regions of Bangladesh [8].

**Figure 1:**
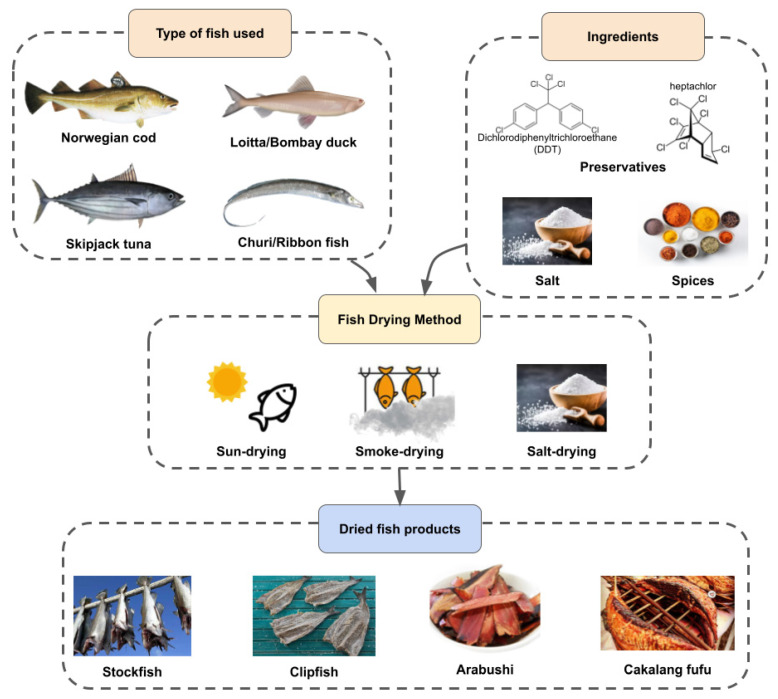
Summary of the types of dried fish, ingredients used and drying methods in a general process of fish drying.

Furthermore, smoke-dried fish is a well-liked food in Asian countries, especially Japan and Bangladesh [9,10]. In Japan, skipjack is smoke-dried to make arabushi, a semi-finished product from katsuobushi, whereby arabushi is yet to be fermented into katsuobushi [11]. The dried fish product is also well known as “cakalang fufu” in Gorontalo, Indonesia [11]. Several stages of smoke-drying occur in its production for certain enterprises, in which the temperature of the stage’s ranges from around 40 to 90 °C throughout the process [11]. On the contrary, smoke-drying is done by using kilns with firewood in Bayelsa State, Nigeria [10]. Like sun-drying methods, smoke-dried fish are also exposed to the environment, although the fish are smoked in enclosed vessels because of improper packaging and transportation [10]. Overall, dried fish is accepted and consumed all over the globe and produced in varying ways depending on the cultures involved in the product. Hence, the characteristics of dried fish highly depend on such cultures as the type of fish, ingredients used, and drying methods readily affect the characteristics of the product in terms of its nutritional contents and texture. A generalised summary of the types of dried fish, drying method, and ingredients is depicted in Figure 1.

### 1.1. Types of Fish Used for Drying

Various species of fish are commonly used to produce dried fish, which can be seasonal or geographical. Fresh fish degrades quickly; hence, drying the fish is the most common alternative processing technique. In Asian regions such as Bangladesh, South Korea and Japan, dried fish are usually made from iridescent shark catfish (*Pangasius hypophthalmus*), skipjack tuna (*Katsuwonus pelamis*), Alaska pollock (*Gadus chalcogrammus*), Pacific saury (*Cololabis saira*) [12,13,14,15]. Meanwhile, in European countries, such as Portugal, cod species such as *Gadus morhua*, *Gadus macrocephalus* and *Theragra chalcogramma* are used to make salted-dried cod [16]. In addition, the butchering technique applied is also dependent on the type of fish. Generally, the fish will be washed and butchered, before the next step, usually salting or brining. As an example, in the processing of bonito (skipjack tuna) into katsuobushi, the head is cut, the organs are gutted, and the central bones are removed before the flesh is filleted [17]. Meanwhile, the butchering process of the iridescent shark catfish involves beheading the fish, then eviscerating the organs and bones, and finally splitting open from the ventral side in butterfly style [13]. The butchering process is slightly different for production of salted-dried cod. The headed and gutted cods are then partially deboned and split in butterfly style for brine salting [16]. For small fish such as anchovies, fresh anchovies were directly spread and dried [18]. Fresh and clean anchovy may not need gutting or heading as it is not salted, like the other aforementioned dried fish products. Still, the butchering process is needed to clean medium and large fish, which can also prevent pungent fish odour.

### 1.2. Dehydrated Fish and Fish Products

Dehydrated fish is generally determined by the decrease in water activity (a_w_), which subsequently decreases or inhibits the microbial activity in the fish, while minimising the deterioration of the fish itself [19]. Fish are usually dehydrated as a means of preservation, as fish is well-known for its perishability. The products that stem from the dehydration of fish are categorised under an umbrella term, dried fish. In reality, dried fish products vary according to the type of fish used, the drying method, and the ingredients used during the whole process. As a result, the physicochemical, microbial, biochemical and safety characteristics of fish change after it is dried, along with its sensory attributes that mainly refer to the market preference.

### 1.3. Drying Conditions and Their Roles in Fish Quality

As discussed previously, fish are widely dried by salting, smoking and especially sun-drying, depending on the cultures and preferences of people around the world. While the end goal is to dehydrate and preserve the fish, certain conditions must be met to ensure the quality of not only the product but also the process of drying fish. Factors such as temperature, drying environment and time can affect the outcome of the dried fish product in terms of safety and preferability of the product. Generally, the fish are dehydrated by lowering the water content of the fish. This is obviously seen in sun-dried fish, especially in Bangladesh [6], where fish are laid out in the sun to be dehydrated. The high temperatures in Bangladesh dry fish efficiently but less hygienically if the fish are exposed to the environment during drying. To prevent spoilage during drying, studies have proposed to dry fish in a convective fish dryer at high temperatures, initially at 90 ℃ and then 60 ℃ to optimise the drying time and final water content in the fish [20]. Methods like these ensure that fish are dried in an enclosed and protected environment, keeping the products out of reach from harmful microorganisms, insects, and unpredictable forecasts that might damage the dried fish products. In fact, modern methods of dehydrating fish like freeze-drying, convection, and vacuum drying all involve enclosed and sanitary environments.

Another notable factor to consider when drying fish is the method of butchering. Interestingly, there are various ways fish are butchered depending on the culture and how the fish is to be dried. Some fish are gutted vertically; some use lengthwise dorsal cuts, otherwise known as the “butterfly” fish; and others involve the stripping of shark meat [21]. According to a previous study, all three of the methods can be found in Panama while only the latter two are found in southern Sinai [21]. The method of butchering is vital for efficient salt drying as specific cuts can be made on the fish to ensure that even amounts of salt enter the fish. As an example, long, slanted cuts are made on both sides of the fish to facilitate salt penetration when fish are gutted vertically to be dried in Panama [21]. This helps to dehydrate the fish osmotically at parts where the fish meat is thicker.

### 1.4. Health Benefits of Dried Fish

**Figure 2:**
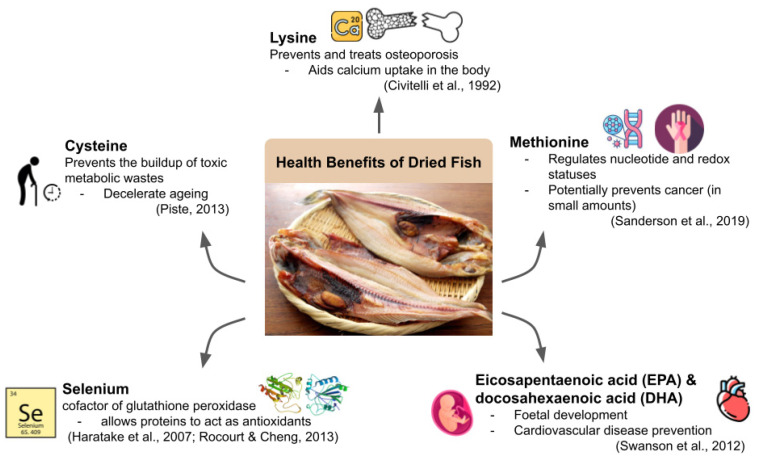
Summary of health benefits of dried fish constituents such as lysine [28], methionine [27], EPA and DHA [29], selenium [30,31], and cysteine [26,28].

Dried fish is widely consumed not only for enjoyment but also for its nutritional values and health benefits. Fish itself is known for its richness in proteins, healthy fats, and minerals. These properties are also well preserved in dried fish products, furthering the benefits by prolonging the shelf-life of the fish by drying them. In a past study, it was found that the protein content of sun-dried fish ranged from 49.23–62.85%, depending on the used type of fish [22]. According to that study, snake-headed fish (*Channa striatus*) charted the highest protein content (62.85%), while a type of catfish (*Wallago attu*) contained the least (49.23%). As illustrated in Figure 2, essential amino acids absent in either plant or meat proteins like cysteine (28 to 25 mg/g), methionine (0.18–2.66 g/100 g) and (0.89–9.864 g/100 g) lysine were found in dried fish [23,24,25]. It is found out that cysteine and methionine are effective antioxidants in which cysteine prevents the build up of toxic metabolic wastes that accelerate ageing whereas methionine regulates nucleotide and redox statuses [26,27]. Additionally, it was stated that methionine metabolism could also be linked to tumour cell metabolism, making methionine possibly essential for cancer prevention [27]. As for lysine, one study that claims that L-lysine could have preventative and therapeutic effects on osteoporosis as lysine aids in the uptake of calcium in the body [28]. It mentioned that dried fish proteins contain essential amino acids for body growth, repairing functions and metabolism [23]. Hence, it can be concluded that the protein contents in dried fish aid in regulatory functions in the body and prevent various diseases (Figure 2).

The fat contents in dried fish are claimed to be healthy, especially when dried fish have lipid oxidation properties by omega-3 polyunsaturated fats (PUFA) [32]. For instance, eicosapentaenoic acid (EPA) and docosahexaenoic acid (DHA) are long-chained omega-3 fatty acids that help in foetal development and the prevention of cardiovascular diseases [29]. Dried fish has been declared to be rich in calcium, phosphorus, and β vitamins, which aid in bone development and maintenance [5]. Another notable mineral present in dried fish is selenium, which is a nutrient that allows proteins to act as antioxidants and anti-inflammatory substances in the immune system by being the cofactor of glutathione peroxidase [30,31]. Since selenium was previously considered a toxin [31], moderate amounts of selenium should be taken to maintain the necessary levels. It was also concluded that niboshi, a type of dried anchovy product in Japan, was sufficient to sustain the dietary intake of selenium in Japan in which little to no deficiency cases were reported, at least back in 2007 [30]. Usually, dried fish is salted as part of the drying process to both hasten the drying process and add flavour to the product. In Sri Lanka, the maximum acceptable level of salt is 12% in dried fish as higher levels of salt could contribute to an increase in blood pressure for consumers [33]. However, studies are being done to reduce and even substitute salt in dried fish to cater to a healthier sodium level intake. For example, potassium chloride and potassium lactate can be used (up to 40%) to avoid the addition of sodium in dried fish, especially for prioritising those with hypertension [34]. Additionally, sodium levels as low as 5% can be used in dried fish, whereby microbial qualities are still maintained [33]. Interestingly, there seemed to be a bias toward lower salt levels (2%) in terms of sensory attributes, which urged future research [33].

In conclusion, dried fish contain various substances that benefit human health. Cardiovascular, bone and immune health have all been demonstrated to be susceptible to improvement via intake of dried fish in moderation. The beneficial contents of dried fish along with the health benefits brought by them are summarised in Figure 2.

## 2. Ingredient Used in Dried Fish and Fish Products

Dried fish has fewer added ingredients than other dried products. Salt is the most common ingredient added to fish for curing and drying. Salted dried fish constituted the types of dried fish available in the market. Despite this, some dried fish are unsalted. Nevertheless, there are other ingredients added to fish, depending on the end product. Generally, these ingredients function to enhance the flavour of fish, increase the safety of the product especially in terms of shelf life, and provide additional health benefits for the product. Therefore, it is vital for safe human consumption.

### 2.1. Salt Added as Ingredient in Dried Fish

**Figure 3:**
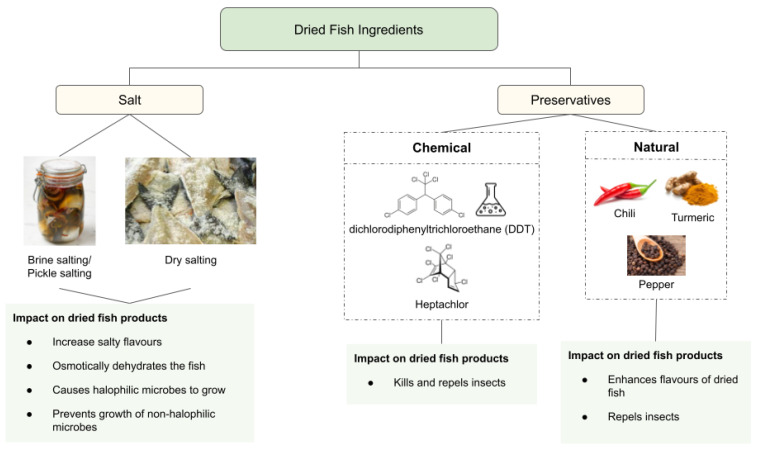
Ingredients in dried fish products and their effects on dried fish.

Naturally, the next step after butchering for salted dried fish is salting. Most locally produced salted dried fish has salt as the only ingredient. The main role of salt in the processing of dried fish is to draw water out of the fish as much as possible, together with the drying step. Dry salting itself can be used as a drying method, as shown in Figure 3, in the production of dried shoal [35]. There are various methods of salting, which include brine salting, pickle salting and dry salting [13,16,33]. This step is crucial to lower the moisture content of the fish, which also means lower water activity, a_w_. Lower a_w_ has been shown to reduce the microbial activity on the fish, which can prolong the shelf life of the fish. It has been found that as the salt concentration increased, the water loss also increased salted sun-dried fish [16]. Normally, a high amount of salt up to 40% is used to cure the fish, but recent studies proposed ways to reduce the amount of salt diffused into the final product. A low percentage of 5% salt in dry salting has been shown to cause low microbial count to the dried fish, which is desirable compared to the average of 30% salt commonly used in Sri Lanka [33]. Nevertheless, other methods of salting such as brine salting and pickle salting implemented higher ratios of salt, but the draining process was done afterwards [16,36]. Some dried fish products do not require a salting process, such as in Indonesian dried anchovies [18,37].

### 2.2. Additives in Dried Fish

Other than salt, additives are added for preservation and adding value to the dried fish, as shown in Figure 3. For preservation, chemical and natural preservatives are applied. However, the chemical preservatives are usually harmful and have been banned in the countries it which they are used. They include dichlorodiphenyltrichloroethane (DDT) and heptachlor, which are harmful organochlorine pesticides used to combat insect infestation of the dried fish products, prominently in developing countries [38]. There are other potential methods to prevent flies and microbial activity during the drying process such as nitrite, acid spray and dip method, and phenolic compounds [39,40]. In addition, spices such as turmeric, chilli, and pepper have been added to the dried fish, as shown in a study [33]. Even though the addition of spices added value in terms of sensory qualities of the dried catfish, the microbial count was still high and further research is needed to improve the process. Hence, salt is the main constituent of non-fish ingredients, but other additives can be added, depending on its function.

## 3. Changes in Physicochemical and Biochemical Characteristics of Dried Fish

Dried fish quality can be assessed through its physicochemical characteristics, such as pH, water activity (a_w_), lipid oxidation, proximate composition, and colour. The changes of these parameters in terms of salting and drying are discussed in this review. Evaluation of the dried fish physicochemical and biochemical properties is vital to ensure that each parameter is at a level that causes low microbial load. Furthermore, the salting and drying processes influence these parameters greatly, where it shows the supposed changes and levels that are suitable for consumption and storage (Figure 3).

### 3.1. Physicochemical Changes in Dried Fish

#### 3.1.1. pH

The evaluation of pH of dried fish products can be an indicator for various parameters in the production of dried fish itself. Generally, different dried fish species have varied relationships between flavour and pH. Nonetheless, dried fish with near neutral pH are considered more palatable than quite acidic or quite basic dried fish [41]. The salting and drying techniques can influence the pH of the dried fish, where this pH can also affect the microbial load of the dried fish. Hence, it is a good indicator of the degree of spoilage. In salting process, it is found that salted sun-dried fish cause lower pH than unsalted sun dried and kiln dried fish, as shown in the drying of *Pangasius hypophthalmus* [13]. This is potentially caused by the denaturation of protein by the addition of salt to the fish before and during the drying process. Meanwhile, in a study to evaluate 10 dried fish in India, the pH of the dried fish samples ranged between 6.23 and 8.33 [42]. These dried fish are traditionally processed and were obtained from dried fish markets, which are usually the open-air type. The lower pH in salted dried fish is potentially due to the production of acidic compounds, and vice versa [43]. Therefore, salted dried fish has lower pH than unsalted dried fish, regardless of the salting techniques.

In terms of drying methods, the common methods include traditional sun drying or cabinet and tunnel sun drying, mechanical drying using barrel kiln and smoke drying. It has been shown that the smoke drying of fish without salting caused an increase in pH, from 6.2 in fresh flying fish to 6.8, after smoke-drying [44]. Meanwhile, kiln drying caused declined pH, from 6.75 to a range of 6.12–634. Despite this, the same study showed an increase in pH in the sun-dried fish, without the addition of salt [13]. Meanwhile, in another study by Farid et al. (2014), the salted sun-dried fish showed a decrease in pH, from 6.9 to 6.2 during sun drying but higher pH of 8.3 along the storage period. Other than these common techniques, freeze drying of fish can has also been applied to obtain and preserve fish products. The freeze drying of fish mince without additives caused no significant pH change, where the pH of wet fish was the same as the freeze-dried fish, which was at pH 6.7 [45]. Hence, the drying methods do have impacts on the pH of the fish, but the addition of salt has a greater effect on the pH of dried fish. The combined effect of salting and drying technique is visible, especially in the salted sun-dried fish products. Still, other factors such as the protein and ash content in the fish used may also contribute to the pH alteration.

#### 3.1.2. Water Activity, a_w_

Water activity, a_w,_ of a substance can be defined as the vapour pressure of water in the hydrated substance when compared with saturated vapour pressure of pure water [46]. At the temperature of the food system, a_w_ also can be referred as the ratio of the food system’s water vapour pressure (*P_v_*) (P_a_) to the saturation water vapour pressure (*P_vs_*) (P_a_) [47]. The investigation of a_w_ for dried fish is crucial, as it regulates the microbial load of the products. This is because the microbial and chemical stability of the product are determined by a_w_, during processing and storage. The moisture content of a dried fish product can have a correlation with water activity of the product, although that is not always the case [15]. One study showed that the a_w_ at the end of salting and the end of drying process was similar, even though the water loss was higher during the drying process [48]. Each microorganism requires specific a_w_ to grow on the dried fish. Therefore, controlling this parameter is an important step in ensuring the safety of dried fish. Most bacteria need a_w_ above 0.85 to proliferate, but this is lower for yeast and fungi, where proliferation can occur at a_w_ as low as 0.61 [49]. Almost no microbial proliferation can occur at a_w_ below 0.60. Therefore, it is best for dried fish to have a_w_ in the range of 0.85–0.60.

Dried fish products have relatively low a_w_, as it is the goal of the drying the fresh fish. In the production of katsuobushi, the arabushi form has a_w_ of 0.76, and further processing to karebushi form lower the a_w_ to 0.62 [15]. Another analysis of dried fish products from Indonesia has shown that the a_w_ is between the range of 0.57 to 0.87. The wide range of a_w_ can potentially cause a diversity of shelf life of the dried fish, as chemical deterioration and microbial growth on the dried fish also differs according to a_w_ [50]. The salting process also aids in the reduction of a_w_ in the processing of dried fish. A comparison between unsalted and salted dried fish is sufficient to visualise the effect of salting on a_w_. In various studies, there are difference between the a_w_ of unsalted and salted dried fish, where the salted ones are lower [19,33,48]. Other than salting, advanced treatments can be done to the dried fish products to lower its a_w_ as technology advances in recent years. An example is via the implementation of corona discharge plasma jet (CDPJ) to commercial dried pollock. The freeze-thaw dried pollock or *hwangtae* samples were treated with CDPJ, where the longer the treatment time, the lower the a_w_ became, from 0.61 to 0.36 at 3 min treatment with CDPJ [12]. In brief, the drying and salting process is the main process that should be controlled, so that the a_w_ of the fish can be minimised, such that it hinders most microbial growth and chemical changes.

#### 3.1.3. Lipid Oxidation

Fish contains fats, and these lipids contain highly unstable polyunsaturated fatty acids, which are prone to reaction with atmospheric oxygen. Consequently, this reaction causes the production of hydroperoxide compounds and free radicals, which lead to off flavours and odour in dried fish, also known as oxidative rancidity [51]. In dried fish, which is a muscle-based food, lipid oxidation can cause a significant degradation of the flavour, colour, nutritional value, and texture [52,53]. Therefore, evaluation of the lipid oxidation is vital in the quality assessment of dried fish products. Hence, the most common method to identify level of lipid oxidation applied in previous studies is by measuring the products of lipid oxidation itself, which includes hydroperoxide and malonaldehyde (MDA). Hydroperoxide is the primary product of this oxidation, which can be empirically measured as the peroxide values (milliEq/O_2_ kg). The consequent reaction involving hydroperoxide converts it into MDA, which is one of the secondary products. Thiobarbituric acid-reactive substances (TBARS), which are expressed in mg MDA/kg, enable the measurement of the amount of MDA [51,54,55].

In dried fish, several factors affect the lipid oxidation of the fish products. Studies have shown that the TBARS value is directly proportional to the drying and storage period [13,51]. The fresh fish before the processing into dried fish has significantly lower MDA/kg than the dried form. Here, the drying temperature also affects lipid oxidation of the fish, where higher drying temperature, from 50 to 70 °C, cause less oxidation of lipids in fish [13,56]. Moreover, storage period also has significant impact to the lipid oxidation of dried fish products. Oğuzhan Yildiz’S [51] study emphasises the effect of storage time to the lipid oxidation, regardless of the treatments done to the dried fish. The result indicates that the longer the storage time, the higher the TBARS value, which means higher oxidation of lipids in fish. The explanation behind this phenomenon might be that the longer the dried fish is exposed to atmospheric oxygen, the greater the contribution to the increase of lipid oxidation during processing and storage [56]. It is important to note that these studies were conducted on one species of fish each, which means the lipid content of the fish was constant. In brief, the TBARS value is an extensively used indicator to evaluate the quality of the dried fish, after the drying process and storage period.

#### 3.1.4. Colour

The colour of the dried fish is an important visual indication of quality, and it will be one of the early factors that contributes to product acceptance. Thus, a quantifiable unit to measure the changes is required to analyse the quality of the dried fish. The colour assessment is done via the quantification of colour, by implementing the CIELAB colour scales (L*, a*, b*). The scale is based on CIELAB uniform space, where the measurement of colour coordinates a* (redness) and b* (yellowness), together with the psychometric index of lightness, L* (lightness) are carried out [57]. Notably, different colour in dried fish can be deemed desirable, depending on the fish species. In the assessment of dried fish, the value of L*, a* and b* parameters are compared before and after drying. This way, the influence of each process in the making of dried fish can be observed. Based on previous literature, the significant changes were usually detected in the L* and b* values after drying. In a comparison between hot air drying, freeze-drying, sun-drying, and solar conduction drying, the most browning was observed in traditionally sun-dried fish samples, followed by hot air drying, solar conduction drying, and lastly freeze-drying [58]. This observation was based on the highest value of b* in traditionally sun-dried fish. The suggested contributor is the non-enzymatic browning events like the Maillard reaction and protein-lipid oxidation reactions, which are facilitated by high temperatures [55,58]. Another study where yellow croakers dried using hot air dryer showed a higher b* value than that of low temperature vacuum drying and freeze drying [59]. The observation implied that higher drying temperature contributed to more browning of dried fish.

A comparison of the use of microwave vacuum drying technique to hot air-drying technique to dry mackerels has been studied [54]. Based on the study, the results show that although the colour attributes of microwave vacuum drying indicated less browning than that of hot air drying, but it was notable and similar. The same conclusion can be applied to fish-drying via smoke-dryer in barren kiln [44]. The aforementioned study also implied that *Cypselurus cyanopterus* dried via smoke drying has desirable traits of good quality dried fish, which was the golden colour of the end product. Nonetheless, the browning of the *C. cyanopterus* in this study was not only contributed by the Maillard reaction, but also via the surface deposition of phenolic compounds during the smoking process. In brief, although higher drying temperatures resulted in more browning or yellowing of the dried fish, which is desirable in most cases, other methods, such as microwave vacuum drying, and smoke-drying are also reliable techniques to obtain similar quality of dried fish. Furthermore, salting is another processing step in the production of salted dried fish. Thus, salting is suggested to influence the colour of the dried fish, due to reduction in water content and presence of minerals in the salt [16]. The lower water content resulted in higher L* and b* values, which can be caused by drying and salting.

### 3.2. Proximate Composition of Dried Fish

Proximate composition involves water content/moisture, lipids, proteins, ash, carbohydrates, as well as vitamins and minerals of the dried fish. By comparison between proximate composition of dried fish processed under different parameters, the changes in nutrient composition of the fish can be observed clearly. Therefore, the assessment of proximate composition is a great tool to evaluate the effectiveness and efficiency of drying methods.

#### 3.2.1. Moisture

Treatments done in the process of drying fish, which usually involves drying and salting, have a great impact on the moisture content of the fish. Nonetheless, it is important to understand the types of water molecules in fish flesh and which one is responsible in the loss of moisture, as mentioned above. In the fish muscle, one of the vital components of the food matrices quality is water. Since these are the only important components in the muscle, there was an inverse relationship between the water and protein contents. In the study of water in fish, types of water can be distinguished such as bound water (intra-myofibrillar) and free water (extra-myofibrillar [60,61,62]. Generally, bound water molecules are trapped in the organised structure of myofibril, whereas free water molecules are located between the space between the protein. Therefore, free water molecules have higher mobility than bound water. This implies that free water is more easily lost during evaporation whilst fish drying; hence, it contributes to the decrease in moisture content [60,61,62]. Various drying methods are implemented to reduce the moisture content of the fresh fish to achieve the appearance and qualities of dried fish. A number of studies have been carried out to compare these drying techniques and identify methods that can lower moisture content more effectively and efficiently, as shown in Table 1. The moisture content of each dried fish decreased significantly after drying. As seen in the table, the preferable moisture content for dried fish, regardless of species, is in the range of 7.0–50.0%. Catfish dried via smoking kiln developed by Nigerian Stored Products Research Institute (NSPRI) has the lowest moisture content at 7.30%, but it requires 15 h of drying time achieve the moisture level. Generally, in dried fish, faster drying time requires a higher drying temperature, as seen in the usage of electric oven [13,63,64]. It only needed 30 min at 120 °C to lower the moisture content of catfish from 71.85% to 15.62% [64]. Nonetheless, drying via microwave vacuum oven can achieve the similar effect as high temperature drying. In a study by Viji, Shanmuka Sai, Debbarma, Dhiju Das, Madhusudana Rao and Ravishankar [54], a reduction in the moisture level to 31–34% was achieved in only 1.2 h via microwave vacuum oven compared to 12 h needed for hot air drying.

Sun drying is the traditional method of drying fish, and it has been proven to be able to reduce the moisture level. Despite this, the contamination by flies and insects during open drying has become a safety concern, as some people have used organochlorine chemicals as insecticide [38,53]. One of the solutions requires the usage of a thick mesh of nylon or a mosquito net while the fish were drying in the sun to protect them from contamination from the outside, bird attack, and fly infestation [43]. Fish can be dried using a smoking kiln, which is also widely used in fish drying [13,64,65,66]. In terms of moisture content, this technique can lower the level, as low as 15.30% in dried tilapia, at a lower temperature compared to ovens. Even so, the drying time needed is typically in the range of 10–24 h, longer than higher temperature drying methods [64,65,66]. Other methods such as hot air and freeze dryings have been implemented, which also show a desirable result in the reduction of moisture content in dried mackerel and yellow croaker [54,59]. Other than drying, salting also aids in the lowering of moisture level of fish. Curing shoal via dry salting caused a 28.19% reduction of moisture content in a 7-day curing period at ambient temperature [35]. The amount of water lost was significant and comparable with other drying methods. In another study, iridescent shark catfish were sun-dried. One batch was salted and the other was not. The moisture content in both batches was successfully reduced, but there was no clear distinction regarding the effect of adding salt combined with heat from the sun [13]. Regardless, the combined effect of drying and salting or brining may cause better rate of water loss, as seen in the sun drying of shoal and shark catfish compared to dry salting alone [13,35,43].

Hence, the process of salting and drying the fish influenced the moisture content greatly. A diverse range of drying methods can be implemented to dry fish, where each method has its own advantages and limitations. Pre-treatment via salting or brining may supplements the drying process to a certain extent, which means water loss can be accelerated [33]. Nonetheless, the optimisation of several of the drying techniques needs to be undertaken, such as in dry-smoking and hot air drying, for more efficient drying [35,44].

#### 3.2.2. Lipid

Due to various factors, the nutritional composition, hence, lipid contents of fish vary diversely. These factors include variations between fish species, ecological conditions, fishing season, size, reproductive status, and aquaculture feeds [43,67]. Moreover, fish can be categorised based on their lipid content, where fish with more than 5% lipid content is considered as lean fish, and fatty or oily fish has more than 5% lipid [65]. Still, fish is considered as a good source of fat, where critical dietary fatty acids such as eicosapentaenoic acid (EPA) and docosahexaenoic acid (DHA), which are n-3 polyunsaturated fatty acids (PUFA) that can be obtained by fish consumption [67,68]. Therefore, it is vital to analyse the crude lipid content of the fish, so that recommendation of appropriate intake of nutrients, in this case, lipid, is in the range of the daily requirement that can be given, and the best packaging method can be determined for longer shelf-life. The measurement of crude or total lipid in fish is part of the proximate composition analysis, which means it is represented in percentage. Generally, the drying process in the making of dried fish caused a significant increase in the lipid content, based on the studies referred to in Table 1. Despite this, in a study by Chukwu and Shaba [64], drying the catfish by using an electric oven was able to retain more lipid than with a smoking kiln. This may be caused by the enhanced fat exudation, paired with the moisture evaporation in the slow process and extended exposure to heat in smoke-drying [64,65]. However, lower levels of lipid after drying can also be beneficial in terms of longer shelf-life. There is a lower chance of rancidity of the dried fish caused by lipid oxidation if the lipid content is low to begin with, hence low levels of free fatty acids (FFA) [35]. Furthermore, the lipid content showed a significant reduction after storage, a contributor to which might be oxidative deterioration of lipid, affecting the extraction of the lipid [43].

Regarding dried mackerel, the fish is considered as a fatty or oily fish [67], which causes it to have a high degree of unsaturation [54]. Hence, during thermal treatment, the mackerel can be highly susceptible to oxidation. Although drying methods have little effect on the lipid content of the dried fish, the combination of hot air and decrease in water activity may decrease the lipid, e.g., in shark catfish, the rate of lipid hydrolysis is higher at higher temperature. The study suggested that this can happen due to the longer drying time using a smoking kiln, which may cause more lipolytic enzymatic reactions to occur, releasing more FFA [13]. Nonetheless, there may be a relation between lower lipid content in dried fish with the salting process. In the drying of shoal and shark catfish, the ones that have been salted either by dry salting or brining, showed significantly lower fat content [13,35]. This can be caused by the increase of lipolysis, up to 4% with higher concentration of salt, as shown in the production of Chinese dry sausage [69]. Therefore, although drying techniques generally do not have significant effect on the lipid content of the dried fish, the addition of salt may reduce the lipid level, due to lipolysis of crude fat.

#### 3.2.3. Protein

Fish is generally known as a rich source of protein. Fast skeletal (ordinary) muscle and slow skeletal (dark) muscle make up most of the muscle arrangement in fish [70]. The major muscle proteins in fish include myosin, actin, tropomyosin, troponin, paramyosin, titin, α-Actinin, parvalblumin, and myoglobin [71]. Sixty percent of the total mass of skeletal muscle protein is composed of myosin. Meanwhile, in aerobic muscle cells, myoglobin is typically present in high concentrations in the sarcoplasm. Muscle colour is significantly influenced by changes in this protein, primarily the redox state of the heme iron, as the redness of muscle is inversely correlated with myoglobin concentration [71]. Thus, with the abundance of protein in the fish muscles, it has been suggested to be consumed as a meat substitute, similar to plant-based protein, as it has less significant environmental impact than red meat [72]. In the studies of fish drying, it has been observed that the drying process caused the protein content in various types of fish to significantly increase compared to that of the fresh fish. Several research papers have suggested that this is due to the retainment of protein nitrogen during drying [35,65]. Furthermore, Farid, Latifa, Nahid, and Begum [43] proposed that the significantly higher protein after drying may be caused by Maillard reaction and alteration of pH whilst drying. Another explanation is that this may be caused by the dehydration of water molecules that present between protein molecules, which caused the aggregation of protein [13,59]. Although the protein content of fish surged after drying, it plummeted after several months of storage in ambient temperature, as shown by Farid, Latifa, Nahid and Begum [43], in shoal drying. Therefore, storage conditions and packaging methods should be optimised to prolong the shelf life and maintain the quality of the dried fish. Vacuum packaging, active packaging approach via oxygen absorbers, composite materials such as Paper-Polyethylene (P_a_P_e_,), Polyethylene-Paper (P_e_P_a_) Polyethylene-Paper-Polyethylene (P_e_P_a_P_e_) are several packaging methods that can be implemented for this purpose [73,74]. To add to this point, a study by Islam, et al. [75] observed that 9 in 10 consumers of dried fish in Dhaka city, Bangladesh agreed with the price increase for better packaging.

As for the effect of salting to the protein content of the dried fish, it has considerable effect on the dried fish, such that the salted sun-dried fish has lower protein level than unsalted sun-dried fish [13]. This decrease in protein content also occurred after storage of the dried shoal [43]. This affect can be ascribed to the addition of salt in the dried fish, which caused protein in the fish muscles to swell, where the protein consequently denatured. In the drying of yellow croaker, low temperature vacuum drying contributed to the highest fish protein content post drying, compared to freeze-dried and hot air-dried fish [59]. The extreme temperature in freeze drying can cause cold denaturation of protein, where at sufficiently low temperature, unfolding occurs and the internal polar groups of protein are then exposed to water [76]. Moreover, prolonged exposure to high temperature treatment in hot air drying can be another reason for the protein to denature, hence the lower protein content after drying [77]. In addition, longer drying time in sustained heat can cause the denaturation of protein [59,64].

In brief, several factors can influence the protein content of the fish during and after drying, and post storage. Temperature, addition of salt, storage conditions, and packaging methods are several reasons to the fluctuations observed in the protein content along drying and storage. Therefore, with this knowledge, more optimised drying methods such as low temperature drying, smoking kiln and electric oven drying. Moreover, active packaging systems via oxygen absorbers can be applied to improve quality and food safety.

#### 3.2.4. Ash

Ash is described as the inorganic residue left over following either full oxidation or ignition of the organic materials in a food sample [78]. Ash content is a proximate composition which is usually analysed in the quality assessment of food products. To analyse the ash content, the fish is incinerated, the organic matter is removed, and the remaining inorganic matter is white ash [13,78]. Ash analysis is vital as it is the measurement of the mineral content of the food. Hence, it serves as the initial step in preparing a food sample for a particular elemental analysis, such as one for highly dangerous heavy metals or for essential nutrients [79]. In general, the ash content of dried fish surged after drying, and addition of salt enhanced this increase, as shown in Table 1. When comparing the salted dried fish with the unsalted ones, ash content is higher in salted dried fish. Also, fish that was sun-dried in the open space has been shown to have higher ash content than those dried in a closed system. Therefore, the increased level of ash in dried fish can be caused by salt content and exposure to open air. Several studies have implied that the higher ash contents were caused by substantial water loss due to the presence of salt, which enhanced the amount of water drawn out by the drying process itself. Hence, dry salting of shoal shown significantly higher ash content, comparable to sun-dried shoal [35,43]. To further prove this point, the ash content of salted dried shark catfish, 18.72%, is notably higher than unsalted dried fish, 5.22% [13].

Moreover, the higher ash content of some of the dried fish may also be caused by the drying conditions. By comparing the drying techniques of various studies, fish dried in the open, mostly sun-dried fish, has higher ash level post-drying than methods involving closed systems, such as freeze drying, kiln drying and electric oven drying [43,59,64]. The open drying in sun-dried fish allowed dust carried by the wind, insect, and bird infestation to settle on the fish, which caused the increase in organic matter. Consequently, dust and foreign matters accumulated on the fish, thus, the higher ash content [13]. In conclusion, the drying process, which includes salting and drying condition, can contribute to higher ash content.

#### 3.2.5. Carbohydrate

Interestingly, there are not many studies focusing on the carbohydrate content aside from the percentage of carbohydrates in dried fish. As seen in Table 1, the carbohydrate composition in dried fish is generally much lower than the other components. Carbohydrates are generally present in small amounts in fish [80], as proteins are usually the more emphasised nutritional value in dried fish. Nevertheless, carbohydrates are still required as a nutritional dietary intake. Comparing the smoke-dried catfish by the conventional kiln [64] and the NSPRI-developed kiln [81], it would seem that the NSPRI smoking kiln reduces the carbohydrate composition in dried fish. While this might be a concern for those seeking higher carbohydrate dietary intake, the amount of carbohydrates reduced in the dried fish is not significant (0.98% lesser) (Table 1). For better carbohydrate retention in dried fish, electric ovens could be used, as demonstrated by Chukwu and Shaba [64].

#### 3.2.6. Vitamin

As discussed previously, dried fish consists of various minerals that deem the products beneficial for health. Interestingly, the drying process is also crucial in maintaining the mineral contents of fish. For instance, vitamins can be easily degraded in high temperatures and in sunlight, especially in the case of vitamin A in sun-drying small fish [82,83]. However, this contradicts the findings by Chukwu [64], in which vitamin A content is seen to increase after drying by kiln and electric oven. Putting both statements together, it seems that the degradation of vitamin A is more dependent on sunlight than on high temperatures. This could be compared to a study on photodecomposition of vitamin A by Fu, et al. [84], where vitamin A is seen to be degraded by the induction of light, specifically sunlight (ultraviolet light). Nevertheless, Chukwu [64] suggested that drying fish by electric oven would be better compared to kiln drying due to the reduced fat content and higher vitamin A content after drying. Additionally, the study mentioned that vitamin C was also present in dried tilapia fish and showed no significant changes before and after drying.

#### 3.2.7. Minerals

**Figure 4:**
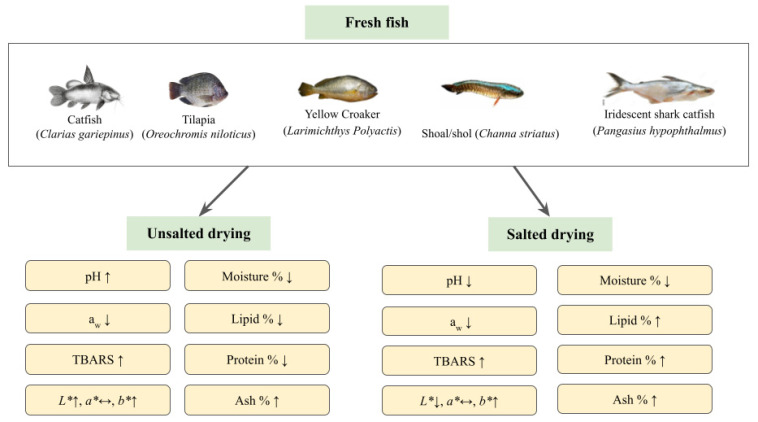
The changes of physicochemical and biochemical properties of various dried fish.

A comparative study by Ako and Salihu [85] showed that smoke-dried fish contained more minerals than oven-dried fish, with lead having the lowest concentration (ranging from 0.46 mg/kg to 1.16 mg/kg) and calcium the highest concentration (0.49 g/kg to 7.55 g/kg). Overall, it was found that there were almost no significant differences in mineral content between smoke-dried and oven-dried fish across five different fish species (*Sarotherodon galilaues*, *Cyprinus carpio*, *Clarias gariepinus*, *Sardinella* spp. and *Labeo* spp.). Despite this, few crucial assumptions were made to make sense of the results of the study. It has been shown that smoke-dried fish are rarely salted, only minimal metal was lost during smoking if any, and that smoke and ash have no effect nor contribution to the mineral content in dried fish [85]. In addition, iron, potassium, sodium, and phosphorus were also found in dried fish [23]. Iron, phosphorus, and calcium existed in varying amounts depending on the type of fish used, but the composition range of each substance was consistent. Similar to the previous comparison, calcium (2.48–2.54 g/kg) still had the highest concentration in dried fish while iron (0.043–0.184 g/kg) had the lowest, with the concentration of phosphorus (0.94–1.91 g/kg) in between [86]. As mentioned previously, salt is added to fish as part of the drying process. As such, sodium from salt would also contribute to the minerals found in dried fish in concentrations up to 40%. In the case where lower sodium levels are preferred, potassium is used to replace sodium [34]. Figure 4 summarises the changes of physicochemical and biochemical properties of various dried fish.

## 4. Sensory Characteristics of Dried Fish

The sensory characteristics of a product can be assessed using a testing technique based on the sensing process, physio-phycological process, or recognition of the human senses as the main appliance for determining its acceptability [87]. Sensory characteristics can be measured via colour, odour, texture, flavour, appearance, palatability, insect invasion, and overall acceptability on the products (Table 2). Measuring sensory characteristics in fish drying are crucial in order to determine and maintain the quality of the products. A sensory score of a dried fish has been suggested to indicate the better quality of the products. Fish dried using solar tunnel drying method showed the most desirable overall acceptability score [88]. Fish dried using traditional sun drying method obtained the least desirable sensory score, and exhibited a poor colour, texture, and odour quality compared to solar tunnel drying. Meanwhile, the fish dried using improved drying method obtained a more desirable overall acceptability sensory score. Apart from that, three different drying methods, sun, oven, and smoke drying, were applied on three different fish samples, *Bonga* spp., *Sardinella* spp., and *Heterotis niloticus* [89]. From the study, it was found that the sensory evaluation for the best taste was recorded for fish samples that were dried by smoke and oven drying. In addition, the most appealing dried fish sample was recorded from the smoked samples and the least appealing dried fish sample was recorded from the sun-dried samples.

Application of different types of dryers on the fish drying also affects the sensory characteristics of the dried fish products (Table 2). Among the different dryers used, which were solar tray, solar cabinet, solar tunnel, and electrical dryers, it was found that the most desirable overall acceptability score was obtained from the electrical dryer [90]. The controlled conditions in the electrical dryer, which are operated at 60 °C temperature and 75% corresponding relative humidity, influenced the highly satisfactory quality of the dried fish products. Prolonged drying may contribute to the growth of fungal and discoloration, which were observed after drying the fishes under a solar tunnel dryer [90].

**Figure 5:**
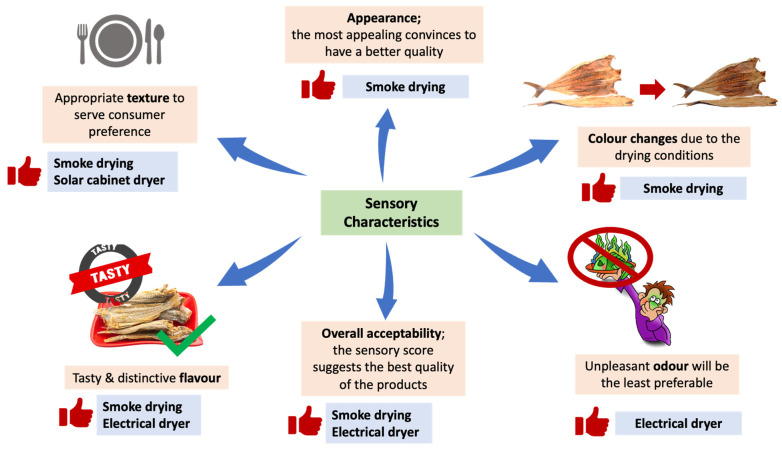
Sensory characteristics of dried fish with preferable parameters.

Other than that, the sensory characteristics of dried fish changes when the fish is osmotically dehydrated by sugar beet molasses [91]. The use of sugar beet molasses is non-conventional in the dried fish industry, and it is suggested for future research because studies showed better water loss from fish meat when including sugar beet molasses in the osmotic dehydration of fish [91,92]. However, this method of fish drying is less preferable due to the drastic sensory changes in aroma and taste [92]. It was found that the aroma and taste of the dried fish should be improved if the sugar beet molasses-dried fish is to be commercialised (Figure 5).

## 5. Microbial Characteristics of Dried Fish

One of the most concerning factors when it comes to the edibility of dried fish is whether the microbial constituents in the products are at safe levels. This of course is highly dependent on the method of drying and the extent of dryness the fish is able to reach. Additionally, the ingredients added to the dried fish also play an essential role in managing the microorganisms in dried fish, as does the drying environment the fish is exposed to. In the case of sun-drying, the microbial characteristics of dried fish change due to the exposure of fish to high temperatures and ultraviolet (UV) radiation from the sun. It was revealed that *Escherichia coli* was found in naturally sun-dried fish whereas *Salmonella* spp. and *Vibrio* spp. were completely eliminated by sun-drying regardless of the presence of fish racks [93]. It was also found that fish racks mainly reduced the drying time from 3 to 2 days in comparison to sun-drying without the racks [93]. Nonetheless, sun drying keeps the dried fish products at risk of contamination by other elements like animals and insects since the dried fish are out in the open. As such, natural sun-drying is still less recommended, except when fish racks are used to not only reduce the time and cost of drying but also to minimise the risk of contamination [93]. In an alternate scenario, certain regions of the world are limited to only air-drying when putting out fish to dry in the sun because of seasonal and regional weather conditions. This is seen in Norway, where fish are usually dried in lower temperatures with minimal sunlight [1]. In Nigeria, the use of lower or ambient temperatures to dry fish is also observed as harmattan occurs late into the year, in which temperatures are cooler and wind speeds are higher [94]. This study showed that open-air drying in lower temperatures seemed to yield similar microbial results as in higher temperatures. On the other hand, smoke-drying fish can be said to be less effective at removing microorganisms compared to sun-drying as seen in a past study, where no significant difference in total plate count (TPC) was observed between fresh and smoke-dried fish [10]. However, smoke-dried fish was observed to contain no faecal coliform [10], indicating that smoke-drying could prevent issues in the digestive tract upon consumption of dried fish. *Bacillus, Klebsiella, Staphylococcus, Pseudomonas, Streptococcus* and *Proteus* bacteria could be found in smoke-dried fish, with *Bacillus* spp. being the most prominent (58.4%) at least in *Clarias angularis, Channa obscura and Chrysicthtys auratus* fishes [10]. At the same time, the diversity of fungal constituents of the fish in the study was also reduced by smoke-drying, in which only *Aspergillus, Fusarium and Penicillium* were isolated compared to the 7 isolated fungi strains from fresh fish (Table 3).

For salted dried fish, the microorganisms present are usually halotolerant or halophilic, especially if the microbe is familiar with marine salinity conditions [95]. Not only could the microbes come from the fish itself, but also from salt. The presence of *Bacillus* spp., *Micrococcus* spp., and Coryneform bacteria in salt could be the cause of dried fish spoilage, especially when the fish is salt-dried [95]. Several fungi genuses like *Aspergillus, Rhizopus, Penecillium, Absidia* and *Mucor* are also found in salted fried fish as they possess halotolerant or halophilic properties [95]. From this, it can be concluded that mere salt-drying is inefficient to eliminate harmful microorganisms from dried fish products. As such, salted fish are normally sun-dried afterwards to achieve lower levels of water content and remove harmful microorganisms. More scientific methods of drying fish have been developed to hasten and improve the quality of dried fish. A critical bonus factor of modern drying methods is that dried fish can be beamed relatively safe from harmful microorganisms. Comparing solar conduction drying, hot air drying and freeze-drying techniques to traditional sun-drying, the total viable count (TVC) and total fungal count (TFC) of all modern techniques charted lower values [58]. Once again, the findings of the study emphasised the lack of hygiene while sun-drying fish because of the unenclosed drying environment of the fish. Nonetheless, Table 3 shows that the number of microbes found in fish dried by advanced technologies are generally not lower than the other traditional methods. The quantification of microbes highly depends on the type of fish used and the surrounding environment, which differs from study to study. Alternatively, fish that are osmotically dehydrated with sugar beet molasses showed lower concentrations of bacteria (4.23 × 10^4^ CFU/g) than with a salt and sucrose aqueous osmotic solution (7.33 × 10^4^ CFU/g) [91]. Although the TVC in dried fish from this drying method still depends highly on the surrounding environment, its potential as a fish drying technique is undeniable and is worth looking into.

To summarise, dried fish is supposed to contain less microbial activity, and certain factors are still able to affect the presence of microorganisms in the products. In fact, some techniques could even draw in microbes from the environment, like with open-air sun-drying and halophile-attracting fish salting. Generally, it can be said that the key to reducing the microbial activity in dried fish would be to decrease the water activity in the product.

## 6. Safety Challenges of Dried Fish and Precautions to Prevent Side Effects

**Figure 6:**
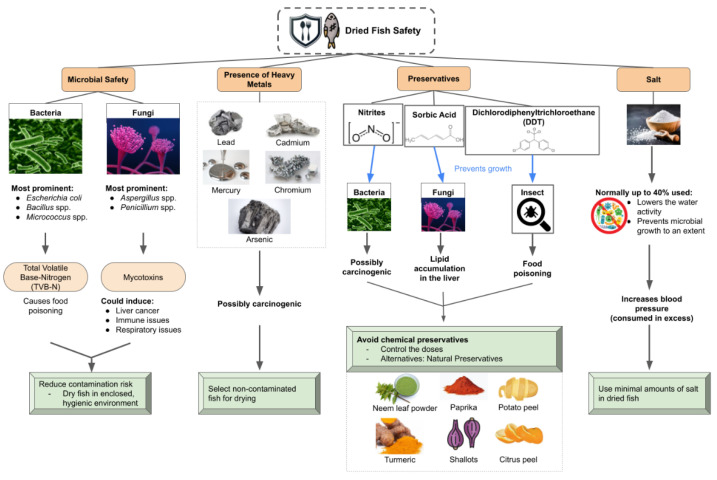
Significant safety aspects of dried fish along with their effects on human health and the prevention recommendations.

While dried fish is widely accepted and consumed by diverse communities, there have always been a few safety concerns circulating the dried fish industry. Fish itself is highly perishable due to its microbial and enzymatic compositions, even under refrigerated and frozen conditions [98]. Although drying the fish aids in prolonging shelf-life, drying alone is unable to fully preserve and protect the fish from hazardous components, nor is it enough for sellers to avoid adding harmful substances to further preserve the fish (Figure 6). As mentioned, the microbial characteristics of dried fish are, albeit lesser compared to fresh fish, still potentially harmful when consumed. The microbial contents of dried fish are also related to the total volatile base-nitrogen (TVB-N) [22]. TVB-N are biogenic amines that could be formed when foods are stored [99]. Excessive consumption of these biogenic amines can cause health issues like food poisoning in humans [100]. These biogenic amines are formed from the microbial decomposition of fish [101], explaining the relation between thriving bacteria and high concentrations of biogenic amines in a study by Mansur, Rahman, Khan, Reza, Kamrunnahar, and Uga [22]. Thus, it is extremely important to ensure that dried fish products are not contaminated, especially when toxic components could be released (Figure 6).

Furthermore, fungi should also be taken note of when avoiding contamination of dried fish. This is because harmful mycotoxins can be produced from fungal contaminations. From a study by Deng, Wang, Deng, Sun, Wang, Ye, Tao, Liao and Gooneratne [5], Aflatoxin B_1_ (AFB_1_), T-2 toxin (T-2), ochratoxin A (OTA), and deoxynivalenol (DON) were mycotoxins found to be released by fungi in dried fish products, dominantly by *Fusarium, Penicillium* and *Aspergillus* fungi. If consumed in excess, mycotoxins could cause major health problems like liver cancer, immune issues, and respiratory issues [3]. The study recommended that marketplaces and storage places for dried fish should have better fungal control to prevent mycotoxins from ruining the dried fish products. The challenge for preventing both bacterial and fungal contaminations is that most dried fish sellers do not have the means to afford, nor do they prefer, fish drying by machinery. Because of that, most dried fish are still mass produced by open-air sun-drying, which exposes the fish to contaminants and unpredictable humidity changes in the environment. Hence, it would be difficult to make dried fish completely safe from contamination. However, there have been more studies that focus on making financially friendlier drying methods for sellers. Sufi, et al. [102] were able to create a more economical and efficient drying method compared to the traditional sun drying with a solar tunnel dryer. This development could inspire more people to utilise cheap materials to ensure that safety measures can be taken for the production of dried fish. Other than that, the presence of heavy metals in fish has also been a significant concern for consumers when it comes to dried fish. Previously, lead, mercury, cadmium, chromium, and arsenic were found in dried fish sold in Bangladesh [103]. This was suspected to be caused by the bioaccumulation of heavy metals in aquatic environments [104]. It was found that the amount of chromium itself, along with the sum of other heavy metals in dried fish represent a possible carcinogenic threat in Bangladesh, especially in dried Bombay duck and ribbon fish [103]. Consequently, authorised personnel are pressured to tighten laws on aquatic pollution and regulations on dried fish production to ensure the health of the community [103]. Regarding small businesses, it would be wiser to simply select non-contaminated fish sources for dried fish production (Figure 6).

Like any other commercially packaged food product, dried fish also often contains preservatives. One of the substances used as preservatives for dried fish is nitrites for their inhibitory effects against *Clostridium botulinum*, which could cause food poisoning when ingested [40]. However, there is a limit to the amount of nitrite that can be consumed, especially when nitrites can be carcinogenic when combined with amines or amides [105]. Another commonly used preservative for dried fish is sorbic acid as it is effective against fungal growth, making it a good pair with nitrites since both substances each conquer either bacteria or fungi [40]. However, it was discovered that sorbic acid has adverse effects on hepatic lipid metabolism when consumed in excess, causing lipid accumulation in the liver [106]. Fortunately, there are natural alternatives available to replace chemical preservatives. For example, organic substances such as neem leaf powder and paprika could be used to control insect and fungal contaminations in dried fish [107]. Turmeric, shallots, potato peel, citrus peel, and pomegranate peels have also been said to have protective properties against microbes and oxidation, making them effective preservatives [107]. Hence, there are organic and economically available means of preservation for storing dried fish.

## 7. Advanced Technology in the Progress of Drying Fish and Fish Products

High-quality dried fish and fish products with minimal changes in nutritional and sensory characteristics are highly in demand among consumers. Advances in technology in fish drying are being implemented and explored as a way to serve a safe, fresh, and nutritive products with high quality and minimal changes in dried fish and fish products (Table 4). There are abundant emerging technologies that can be implemented in order to obtain a high quality of dried fish and fish products. For instance, a high-pressure processing, which is a new and novel technology has the potential to significantly increase shelf life of the products with little or no heat treatment [108]. It is a non-thermal process, where the food item to be treated, is put in a pressure vessel that can withstand the necessary pressure, and is submerged in a liquid that serves as the pressure transmission medium. The influence of high pressure on the products quality was evaluated through the sensory characteristics of cod (*Gardus morhua*) after storage up to six months [109]. Moreover, another non-thermal technology, pulse light technology has been implemented for the decontamination of products surfaces and packaging that incorporates of short time high-peak pulses of broad-spectrum white light [110]. This method had been implemented in preservation of fish products which then further analysed for its sensitivity effect on the fish products [111].

Pressure shift freezing assists in reducing tissue deformation and shrinkage of the products. Based on the study conducted by Zhu, et al. [112], pressure shift freezing treated salmon showed that the muscle fibres of the salmon were well maintained. This has convinced us that pressure shift freezing can aid in preserving the quality of fish products. Furthermore, it is known that the success of freezing preservation of fish products depends on the preparation, freezing rate, storage conditions, and thawing conditions. Pressure shifting pressure and pressure assisted thawing that were conducted on sea bass demonstrated a promising result to preserve the products quality [113]. Additionally, pulsed electric field is a processing method that integrates the application of very short electric pulses at electric field intensities [114]. This technology is considered as non-thermal disintegration or preservation process, which is possible for the occurrence of rapid rapture with no effect on the entire cell membrane, depending on the electric field. Study on pre-treated sea bass using pulsed electric field-assisted brining seemed to show a promising technology due to its efficacy processing and preserving fish products [115].

**Figure 7:**
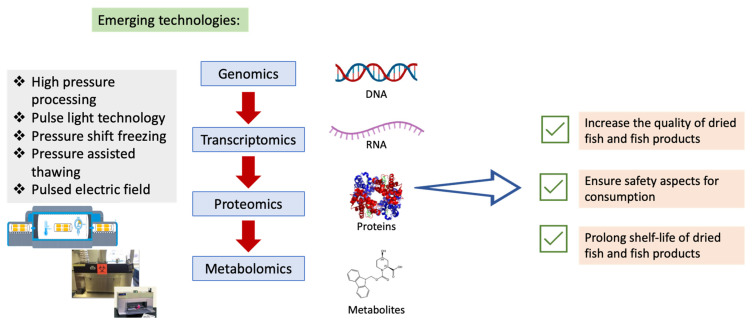
Emerging technologies in enhancing the quality of dried fish products.

Understanding the molecular levels may assist in enhancing the quality of dried fish and fish products. Advancement in omics analysis, which includes genomics, transcriptomics, proteomics, and metabolomics that holistically integrate the study of genes, transcripts, proteins, and metabolites in an organism are useful in understanding the mechanisms that are involved while drying the products (Figure 7). The emerging of technological advancements such as next-generation sequencing technology (Illumina, PacBio and Nanopore) and mass spectrometry coupled with gas and liquid chromatography can provide a high-throughput data generation [116]. The information from the data generation is crucial in determining the best quality of the dried fish and fish products as well as prolonging the shelf-life of the products. Additionally, integrating these emerging technologies may help ensure the safety aspects for consumption.

## 8. Conclusions

Fresh fish is great source of nutrients, and it is easily perishable. Thus, preserving fish is important and drying fish is the most common method, with the added benefits of better nutritional values. Dried fish has higher lipid and protein contents than fresh fish, which is advantageous to fulfil daily nutritional requirements. The richness of dried fish in vital fats and proteins make it suitable for consumption as a meat-substitute, in addition to plant-derived alternatives. Moreover, fish has an array of health benefits; consuming it can be advantageous to prevent cardiovascular disease and osteoporosis, and it is also good for foetal development and as a source of antioxidants. These highlight the importance of fish drying, as an effort to reduce fish waste and optimise its nutritional benefits through preservation. Asian and European countries contribute to the distribution of tonnes of fish, either internally or for export to other countries. Therefore, it is only natural that fish are dried in these regions. Nonetheless, the species of fish to be dried are influenced by season and geographical source. In addition, the method of butchering before the drying process also varies between fish products. Generally, the steps include beheading, eviscerating the gut, and deboning, before filleting or cutting it in butterfly style. Butchering is vital, especially for medium to large fish, to avoid pungent fish odour.

Drying fish involves a number of methods, and hot air drying, microwave vacuum drying, freeze drying, sun drying, and dry salting have been mentioned in this review. These drying techniques require their own set of calculated parameters for the drying conditions, to optimise the fish drying. Parameters such as temperature, drying time, pressure, and salt ratio were studied in the aforementioned literatures to identify the best, most efficient, and effective conditions to dry certain fish. Next, the ingredients added to process dried fish are mainly salt, although other additives, harmful or beneficial, have been seen added to dried fish to prolong its shelf-life. Harmful additives, especially organochlorine pesticides such as DDT and heptachlor were sprayed on the dried fish when it is dried in the open to prevent insects’ infestation of the fish. However, the effects are immensely deleterious to human health; thus, the usage of these pesticides had been banned. Alternatives such as nitrite and acid spray have been implemented steadily to replace the harmful additives. Other additives such as turmeric, chilli, and pepper not only have potential as preservatives, but also enhance the sensory quality of the dried fish.

The drying and salting process certainly leads to changes in physicochemical and biochemical properties of the dried fish, which also depends on the pre-treatment and drying conditions. Hence, analysing these alterations can assess the quality and safety of dried for consumption. Based on the studies, the changes are mainly influenced by the addition of salt, where unsalted drying caused higher pH, decrease in lipid and protein content, and a darker product. Nonetheless, a_w_ and moisture content decreased at different rates, depending on the drying methods and presence of salt as pre-treatment. The lipid oxidation of dried fish increased steadily over time, and can be accelerated during storage, especially in ambient temperature. Thus, packaging methods such as vacuum packing and active packaging via oxygen absorber can be applied to combat lipid oxidation and protein degradation to avoid rancidity. Other than physicochemical and biochemical changes, the sensory characteristics of dried fish are also an important element, as they determine the marketability of the products. More often, the colour, appearance, odour, palatability, flavour, texture, and overall acceptability determine the sensory score of various dried fish. Microbial characteristics of the dried fish are another vital aspect, especially in determining the safety of the product for consumption. The effectiveness of various drying methods to decrease the microbial load to a safe level have been studied extensively over the years. The most common microorganisms that have been detected were *Bacillus* spp., *Aspergillus* spp., and *Penicillium* spp. Open-air sun-drying contributed to more microbial activity than closed system drying methods. Still, there is no one best method in decreasing the microbial activity, the strategy should be focused on lowering the a_w_ of the dried fish, where a_w_ is lower than 0.60 show no microbial proliferation.

The safety aspect of dried fish has been a tremendous challenge, as this industry is made up mostly by traditional drying facilities. The challenges involve microbial safety, heavy metals, and use of preservatives. Microorganisms can harm the consumers via TVB-N and mycotoxins, which induced health issues such as food poisoning and liver cancer. Moreover, bioaccumulation of heavy metals from the fish source is an obstacle, which requires the fish to be sourced from a better environment. Even so, contamination might occur in the new environment; thus, the fishery industry needs to find a long-term solution to this more prevalent issue. In addition, the preservatives itself can be a challenge to the safety of dried fish. When used above its safe threshold, it may be carcinogenic and cause health problems. Hence, natural preservatives such as neem leaf, paprika, turmeric, and shallots have become more desirable to control the parameters that ensure food safety.

Future perspectives for the dried fish industry lie in the cutting-edge fish drying technology and application of omics in dried fish assessment. The advanced fish drying technologies that focus on producing high quality dried fish with minimal changes in quality usually implement non-thermal technology. Furthermore, the advancement in omics analysis is an emerging field to be explored for a better understanding of the quality of dried fish products on the molecular levels. Hence, combining these two emerging technologies may be a leap forward to enhance dried fish safety and its quality.

## Figures and Tables

**Table 1:** Proximate composition of various fish species and its respective drying technique.

Type of Fish	Drying Technique	Moisture (%)	Lipids (%)	Protein (%)	Ash (%)	Carbohydrates (%)	References
Catfish (*Clarias gariepinus*)	Electric oven	15.62	29.60	67.21	3.62	3.84	[64]
	Smoking kiln	28.92	21.20	53.10	3.92	2.78	
	Smoking kiln (developed by NSPRI)	7.30	12.50	68.4	6.40	1.80	[65]
Indian Mackerel	Hot air	31.11	5.28	43.38	17.90	N/A	[54]
	Microwave vacuum	32.45	4.02	44.53	21.61	N/A	
Shoal/shol (*Channa striatus*)	Sun dry, salting as pre-treatment	29.77	5.10	41.48	22.80	N/A	[43]
	Dry salting	48.84	3.99	28.21	18.89	N/A	[35]
Yellow Croaker (*Larimichthys Polyactis*)	Hot air	47.08	14.67	31.32	3.74	N/A	[59]
	Low temperature vacuum	38.56	12.56	41.48	6.26	N/A	
	Freeze dry	47.38	13.43	33.54	4.02	N/A	
Iridescent shark catfish (*Pangasius hypophthalmus*)	Mechanical (kiln) at 60 °C	13.50	15.31	65.16	4.38	N/A	[13]
	Sun dry (unsalted)	14.59	15.17	63.39	5.22	N/A	
	Sun dry (salted)	15.36	9.32	55.53	18.72	N/A	
Nile Tilapia (*Oreochromis niloticus*)	Smoke dry at 60 °C (15 h)	15.30	12.35	49.40	21.61	N/A	[66]
	Smoke dry at 70 °C (10 h)	17.95	7.85	56.70	18.52	N/A	

**Table 2:** The effect of drying methods to the sensory characteristics of dried fish.

Sample	Drying Methods	Parameters						**References**
		Colour	Appearance	Odour/ Palatability	Flavour	Texture	Overall Acceptability	
Glassy perchlet (*Ambassis* spp.) and sole fish (*Cynoglossus semifasciatus*)	Solar tray dryer (65 °C temperature and 75.8% relative humidity)	6.1	5.2	5.4	4.4	7.2	6.0	[90]
	Solar cabinet dryer	5.0	5.9	5.0	5.0	8.1	7.3	
	Solar tunnel dryer (45 °C temperature and 75% relative humidity)	3.6	4.0	3.5	3.0	4.1	3.1	
	Electrical dryer (60 °C temperature and 75% corresponding relative humidity)	6.3	7.8	6.0	7.0	6.0	8.9	
*Bonga* spp.	Oven	Light brown	Moderately attractive	Good	Good	Moderately hard	High	[89]
	Sun	Ash colour	Attractive	Moderate	Poor	Very hard	Moderate	
	Smoke	Fairly black	Very attractive	Very good	Very good	Soft	Very high	
*Sardinella* spp.	Oven	Dark brown	Very attractive	Good	Good	Very hard	High	
	Sun	Brown	Attractive	Moderate	Moderate	Moderately hard	Low	
	Smoke	Dark brown	Very attractive	Very good	Very good	Soft	High	
*Heterotis niloticus*	Oven	Light brown	Attractive	Good	Good	Very hard	Moderate	
	Sun	Grey	Moderately attractive	Moderate	Poor	Hard	Moderate	
	Smoke	Brown	Very attractive	Very good	Good	Soft	High	
	Traditional sun drying	2.81	-	2.38	-	3.27	13.42	Rasul, Majumdar, Afrin, Bapary and Shah [88]
*Hypophthalmichthys molitrix*	Improved sun drying (soaked in salt solution 5%, treated with chilli powder 0.3% and turmeric powder 0.3%)	1.93	-	1.56	-	2.18	6.87	
	Solar tunnel drying (soaked in brine solution, 5% salt)	1.69	-	1.22	-	1.93	5.87	

**Table 3:** Microbes found in dried fish along with their quantities according to their drying methods.

Drying Method	Type of Microbe Found in Dried Fish	Microbes Found in Dried Fish	TPC/TVC/TFC	References
Open-air drying Sun-drying Air-drying	Bacteria	*Escherichia coli*	1.84 × 10^4^/g to 5.3 × 10^6^/g	[22,93]
	Fungi	Fusarium spp. Penicillium spp. Aspergillus spp.	1.00 × 10^2^ to 2.11 × 10^4^ cfu/g 1.23 × 10^3^ cfu/g to 3.67 × 10^3^ cfu/g 1.25 × 10^2^ to 2.40 × 10^4^ cfu/g	[5]
Smoke-drying	Bacteria	Bacillus spp. Klebsiella spp. Staphylococcus spp. Pseudomonas spp. Streptococcus spp. Proteus spp.	4.0 × 10^8^ to 2.30 × 10^10^ cfu/g	[10]
	Fungi	Aspergillus spp. Rhizopus spp. Penicillium spp. Saccharomyces spp. Fusarium spp.	1.0 × 10^4^ to 4.0 × 10^5^ cfu/g	
Salt-drying	Bacteria	Bacillus spp. Micrococcus spp. Coryneform bacteria	6.5 × 10^4^ to 1.4 × 10^8^ cfu/g	[95,96]
	Fungi	Aspergillus spp. Rhizopus spp. Penicillium spp. Absidia spp. Mucor spp.	0.72 × 10^1^ to 1.8 × 10^1^ cfu/g	[95,97]
Hot-air drying	Bacteria	Bacteria Salmonella spp. Escherichia spp. Shigella spp. Fungi: N/A	2.87 × 10^5^ cfu/g	[58]
	Fungi		1.9 × 10^5^ cfu/g	
Freeze-drying	Bacteria		1.90 × 10^5^ cfu/g	
	Fungi		0.63 × 10^5^ cfu/g	
Solar Convection Drying	Bacteria		1.60 × 10^5^ cfu/g	
	Fungi		0.53 × 10^5^ cfu/g	

**Table 4:** Processing methods for preserving the quality of fish products.

Sample	Method	Technology	Reference
Cod (Gardus morhua)	High pressure processing	Non-thermal process with vessel pressure	Matser, Stegeman, Kals and Bartels [109]
Fish products	Pulse light technology	Non-thermal process with high peak pulses	Lasagabaster and De Marañón [111]
Atlantic salmon (Salmo salar)	Pressure shift freezing	High freezing rate	Zhu, Bail and Ramaswamy [112]
Sea bass (Dicentrarchus labrax)	Pressure assisted thawing	Non-thermal process with thawing conditions	Tironi, De Lamballerie and Le-Bail [113]
Sea bass	Pulsed electric field	Non-thermal process with electric pulses	Cropotova, Tappi, Genovese, Rocculi, Laghi, Dalla Rosa and Rustad [115]

## Data Availability

Not applicable.
